# P204: What’s the scope? Pseudomonas aeruginosa outbreak in ICU

**DOI:** 10.1186/2047-2994-2-S1-P204

**Published:** 2013-06-20

**Authors:** S Salmon, M Balm, C Teo, D Fisher

**Affiliations:** 1 Infection Control Team, National University Hospital Singapore, Singapore, Singapore; 2 Microbiology, National University Hospital Singapore, Singapore, Singapore

## Introduction

Surgical Intensive Care Unit (SICU) maintains one bronchoscope for use on its patients. An internal decision to change manual bronchoscope re-processing practices occurred in July 2012. In September and October, four consecutive patients cultured *Pseudomonas aeruginosa* from bronchial washings following bronchoscopy.

## Methods

An outbreak investigation was triggered including analysis of laboratory data, case reviews, investigation of workflows within SICU and sampling of bronchoscope and brushes. No molecular typing was performed as isolates were no longer available.

## Results

Patients 1 and 2 cultured multiresistant *P. aeruginosa* with identical antibiograms. Patients 3 and 4 cultured multisusceptible *P. aeruginosa* and *Serratia marcescens*. Patient 4 also had *Stenotrophomonas maltophilia*. Bronchoscope cultures taken after manual re-processing grew *P. aeruginosa*, *S. marcescens* and *S. maltophilia* with identical antibiograms to Patient 4. Procedure review revealed multiple irregularities including reduction of immersion time in sterilant from 30 to 10 minutes. This change had been adopted at the vendors’ suggestion due to concerns regarding damage to the bronchoscope from exposure to chemical sterilants. In liaison with the Infection Control team, SICU staff devised a new workflow ensuring re-processing of bronchoscopes in an automated washer-sanitiser occurred following use, with sterility checks on the bronchoscope following cleaning.

## Conclusion

Changes to protocols and workflows may have unintended consequences with patient safety implications. Infection Control teams must be alert to the potential for changes in practice of which they are unaware. Maintaining a high profile with ward managers and laboratory surveillance for subtle outbreaks remain important safety nets for patients.

## Disclosure of interest

None declared

